# Anti-Cancer Nanomedicines: A Revolution of Tumor Immunotherapy

**DOI:** 10.3389/fimmu.2020.601497

**Published:** 2020-12-21

**Authors:** Wei Li, Anghui Peng, Huajun Wu, Yingyao Quan, Yong Li, Ligong Lu, Min Cui

**Affiliations:** ^1^Department of General Surgery, Zhuhai People's Hospital, Zhuhai Hospital Affiliated with Jinan University, Jinan University, Zhuhai, China; ^2^Zhuhai Interventional Medical Center, Zhuhai Precision Medical Center, Zhuhai People's Hospital, Zhuhai Hospital Affiliated with Jinan University, Jinan University, Zhuhai, China; ^3^Faculty of Health Sciences, University of Macau, Macau, China

**Keywords:** tumor, immunotherapy, nanomedicine, nanotherapy, review

## Abstract

Immunotherapies have been accelerating the development of anti-cancer clinical treatment, but its low objective responses and severe off-target immune-related adverse events (irAEs) limit the range of application. Strategies to remove these obstacles primarily focus on the combination of different therapies and the exploitation of new immunotherapeutic agents. Nanomedicine potentiates the effects of activating immune cells selectively and reversing tumor induced immune deficiency microenvironment through multiple mechanisms. In the last decade, a variety of nano-enabled tumor immunotherapies was under clinical investigation. As time goes by, the advantages of nanomedicine are increasingly prominent. With the continuous development of nanotechnology, nanomedicine will offer more distinctive perspectives in imaging diagnosis and treatment of tumors. In this Review, we wish to provide an overview of tumor immunotherapy and the mechanisms of nanomaterials that aim to enhance the efficacy of tumor immunotherapy under development or in clinic treatment.

## Introduction

Nanoparticles have become a promising strategy for anti-cancer treatment due to their inherent properties. To date, the clinical approvals for tumor therapy are organic materials, including liposomes (pegylated or non-pegylated) and albumin, while inorganic materials are used for tracking and molecular imaging functions, and only superparamagnetic iron oxide (SPIO) nanoparticles are approved in clinical (1). Nanoparticles for tumor therapy are mainly used for drug delivery, photothermal therapy, modification and preparation of engineered cells, imaging diagnosis, and lymph node (LN) tracing. Normally, nanoparticles are used as a drug-loading platform to improve the efficacy of existed anti-tumor agents. Nanoparticles loaded chemotherapeutic agents and targeting delivery into tumor sites have been applied to clinical treatment (2). However, the tumor immunotherapy based on nanoplatforms still stay at the stage of pre-clinical research. However, it is noteworthy that nanoparticles themselves trigger immunogenic tumor cell death and elicit both innate and adaptive immune responses for tumor control and metastasis prevention (3), which showed a broad-spectrum anti-cancer mechanism.

## Limitations of Conventional Tumor Immunotherapies

Immunotherapy aims to activate immune system to discover and eradicate tumor cells and to inhibit tumor development durable by producing immunological memory (4, 5), while non-specific stimulate immune system directly enhances the reactivity to tumor antigens (6). Until recent years cancer immunotherapies have achieved higher objective response rates (ORR) in patients because of the promotion of T cell function by immune checkpoint blocking (ICB) with monoclonal antibodies (mAbs). Currently, ICB is often chosen as first-line therapy due to the long duration of response in some patients, even after cessation of therapy (**Supplementary Table S1 and S2**) (7, 8). However, there are two major obstacles in tumor immunotherapies, namely the low response rate and the severe immune-related adverse immunotherapy events (irAEs), which are still insurmountable issues in conventional tumor immunotherapy.

### Low Response Rate

Although immunotherapy has shown substantial benefit in treatment of a variety of tumors and exhibit durable response, the majority of patients failed to respond to PD-1/PD-L1 blockers (9–12). 50% patients with PD-L1 positive show tolerance after the initial response to PD-1/PD-L1 blockers, most patients will develop acquired resistance (13). Similarly, Anti-CTLA-4 has low level of ORR (14, 15). Moreover, the ORR of HDIL-2 monotherapy is 10%–19% with complete ORR of 6%–8% (16). Therefore, how to improve the ORR of immunotherapy and how to select appropriate indicators to predict the effectiveness of immunotherapy are the two hotspots of current research. At present, combined therapy is the major way to improve clinical response rate, such as the combination of different ICBs (17–20), the combination of various immunotherapies including tumor vaccines, CAR-T, and IL-2, immunotherapy combined with anti-angiogenic therapies (21) and immunotherapy combined with chemotherapy (12, 22–24). Diverse chemotherapeutics can induce the expression of PD-L1 through distinct mechanisms (25, 26), so the chemotherapy combined PD-L1 inhibitors may produce a synergistic effect. Combining nivolumab with radiotherapy could induce immunosensitization to improve the efficacy of PD-1 blocker (27, 28), while immunotherapy combined with small molecule inhibitors therapies reflects immunomodulatory effects, such as the increase of tumor antigenicity and the promotion of T cell infiltration in tumors (29–31). Overall, given the current clinical projects, except the optimization of the current drug combinative strategies, new platform should be founded to improve the treatment status, reduce side effects and recognize the tumor-specific antigens by engineered lymphocyte.

### Immune-Related Adverse Events

Immunotherapy often results in irAEs (14, 32), whose pathogenesis can be comprehended by the immune-pathophysiology that excessive and systemic immune system activation can occur at any point during treatment course (33–35). Generally, irAEs include skin toxicity, diarrhea, colitis, pneumonia, liver toxicity, and endocrine system toxicity, and the severity of irAEs is divided into 1–4 grades (36). Approximately 30% patients with melanoma who had accepted the CTLA-4 blocking strategy developed to toxicity grades 3–4, suggesting that the inhibitor activated the systemic immune response rather than tumor-specific T cells (14). In most cases, the combined therapy increases the incidence of irAEs (17, 31, 37, 38). Patients with asymptomatic can be carefully observed and followed up. If it cannot reach a higher level stage and relieve the patients’ suffering, withdrawal temporarily or permanently and medical interventions including steroids and immunosuppressive agents are required. Notably, the potential risk of toxicity should not outweigh the overall survival (OS) benefit.

## Applicable Systems of Nanomodified Immunotherapy

As a versatile platform, nanoparticles can not only be loaded with agents to improve their efficacy but also play a role of therapeutic media themselves (**Table 1**). In recent years, nanomedicine has continued to solve existing problems and has achieved the desired therapeutic effects in cancer immunotherapy.

**Table 1:** The types and functions of nanoparticles commonly used in clinical trials.

Particle type	Application in clinical trials	Functions
**Organic NPs**		
Cyclodextrin NPs	CRLX101	improve drug solubility deliver drug to tumor
Liposome NPs	Tecemotide, Lipo-MERIT, DPX-0907, DPX-Survivac, OncoQuest-L, Lipovaxin MM, ISCOMATRIX, AS15, JVRS-100,	improve drug-loading efficiency provide efficient cell affinity increase cellular uptake
Polymeric NPs	CHP-NY-ESO-1, IMF-001, ZYC300	deliver antigen
Solid Lipid NPs	ONT-10 Vaccine	adjuvant
Exosomes NPs	Dex2	extracellular vesicles target and dock to recipient cells stimulate CD8+ and CD4+ T cells
Autophagosome NPs	DRibbles	induce B-cell proliferation and activation induce antibody production induce cytokine secretion antigen cross- presentation
Virus-Like NPs	Melan-A VLPs	restore immune response Induce CTL response antigen cross- presentation
Tumor lysate NPs	WDVAX	activate the immune system
**Inorganic NPs**		
Gold NPs	Colloidal gold	deliver protein biologics

### Drug Delivery Systems

**Figure 1:**
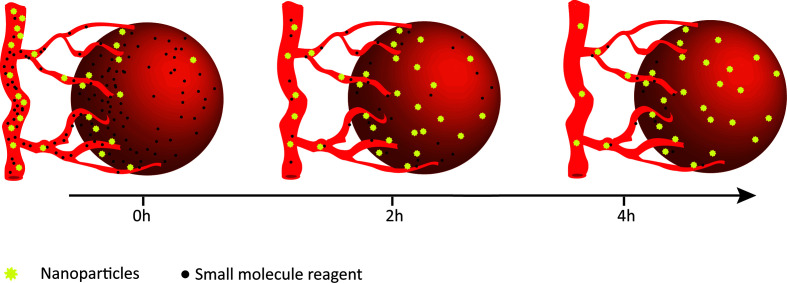
Enhanced retention of nanoparticles. Compared to small-molecule drugs, nanoparticles appear to be gradually enriched in tumors and maintained for a longer period of time.

In terms of ICB and cytokine therapies’ low affinities with the targeted proteins and the obstacle of precise delivery, it is necessary to improve the targeting and the controlled releasing capabilities of anti-cancer drugs to make them work in specific sites and minimize off-target effects (39). Surprisingly, as a drug carrier, nanoparticles increase the biocompatibility and solubility of reagents, prolong their blood circulation time to provide unique advantages in the precise delivery of drugs to target sites (**Figure 1**). Briefly, the nanomodified immunotherapies reduce peripheral toxicity and side effects, making clinical treatment more compliant and precise.

AlbiVax is a novel nanovaccine complex that the antigen is conjugated to Evans blue and can be self-assembled in combination with albumin *in vivo*. Compared with incomplete Freund’s adjuvant, AlbiVax had an almost 100-fold higher efficacy in delivering antigen to LNs, and its ability to elicit immunological memory of peripheral antigen-specific CD8^+^ T cells was approximately 10 times higher than that of incomplete Freund's adjuvant. AlbiVax restrains the growth of various tumors, and the combination of AlbiVax with anti-PD-1 agents Abraxane enhances immunotherapy and eradicates most tumors (40). Encapsulation of anti-PD-1 antibodies with PLGA nanoparticles improves the anti-tumor effect but exhibits higher mortality due to the overactivation of T cells, which can be reversed by reducing dose (41). A multifunctional immunoliposomes named CAT@aPDL1-SSLs promoted the delivery and accumulation of anti-PDL1 antibodies in tumor tissues to activate the infiltration of CD8^+^ T cells at the tumor site with low systemic toxicity (42). In addition to direct drug delivery, nanoparticles package small interfering RNAs (siRNAs) targeting PD-1 and T-cell immunoglobulin mucin 3 (TIM-3) to restore T cell immunity and sensitize the response of cancer cells to T cell killing (43, 44). Loading the PD-L1 trap plasmid into a lipid-protamine-DNA nanoparticle enhanced the local level of PD-L1 trap in tumor microenvironment (TME) but did not induce the appearance of Th17 cells in spleens, indicating that this formulation was better tolerated and had a lower tendency to induce irAEs than the unmodified plasmid (45). Collectively, targeted delivery of agents is based on the enhanced permeability and retention (EPR) effect of nanomaterials and abnormal tumor blood vessels, but the characteristics of nanomaterials have a profound and lasting effect due to their surface modifiability.

### Regulation of the Hypoxic Microenvironment

Hypoxia is a hallmark of TME that induces the tumor resistance to immunotherapy (42, 46, 47). Preclinical and clinical data indicate that hypoxia can significantly reduce the efficacy of anti-tumor immunity (48–51). This mechanism includes the following immunity reactions: a) Hypoxia limits infiltration and proliferation of anti-tumor immune cells; b) Hypoxia acts as an intrinsic immunosuppressive for T cells to inhibit tumor-killing function; c) Hypoxia up-regulates the expression of PD-L1, which promotes the binding of HIF-1α to a transcriptionally active hypoxia-response element (HRE) (50, 52); d) Hypoxia induces high expression of forkhead box P3 (FOXP3), transforming growth factor-β (TGF-β) and CC-chemokine ligand 28 (CCL28) that selectively attracts regulatory T cells (Treg) and increases their functions, which results in antigen tolerance and suppression of the response to effector T cells; e) Hypoxia reduces the production of interferon-γ (IFN-γ) and interleukin-2 (IL-2) by both CD4+ and CD8+ T cells (52). Therefore, reverse the hypoxic TME may potentially increase the immune response.

Nanotechnology has been increasingly used to reverse tumor hypoxic microenvironment (42, 53, 54). Modified nanoparticles could attenuate the unfavorable factors by normalizing oxygen levels through tumor vascular normalization (55, 56), external oxygen delivery, capture and delivery of oxygen from the lungs and generation of oxygen through catalysis of water (47).

Vascular function dictates the efficacy of immunotherapy. Tumor vascular normalization is an important measure to reduce the hypoxic microenvironment (57). Some pure inorganic nanoparticles, including gold or silver nanoparticles, can modulate tumor blood vessels (58, 59). Moreover, many reagents (60) and siRNAs (61) targeting blood vessels can be loaded into nanoparticles to retain vascular normalization (62). In addition, the remodeling of blood vessels increases the enrichment and infiltration of anti-tumor drugs and GrzB^+^ effector T cells in tumors, which sensitize tumor to immunotherapy (63, 64).

Since intelligent nanoparticles respond to TME, they may induce the generation of oxygen. MnO_2_-based nanoplatforms could react with excessive endogenous H_2_O_2_ in TME to generate oxygen *in situ* and to overcome hypoxia limitations for cancer therapy (65, 66). A novel TiO-porphyrin nanosystem (FA-TiOPs) was designed by encapsulating TiO-porphyrin into folic acid liposomes. FA-TiOPs can photosplit water to produce oxygen, which overcomes hypoxia in TME, boosts specific anticancer effects while being harmless to normal tissues, especially under acidic conditions (67). FeSiAuO contains Fe_3_O_4_, mesoporous SiO_2_ and magnetic Au_2_O_3_, which decompose into O_2_ in TME under light irradiation (68).

**Figure 2:**
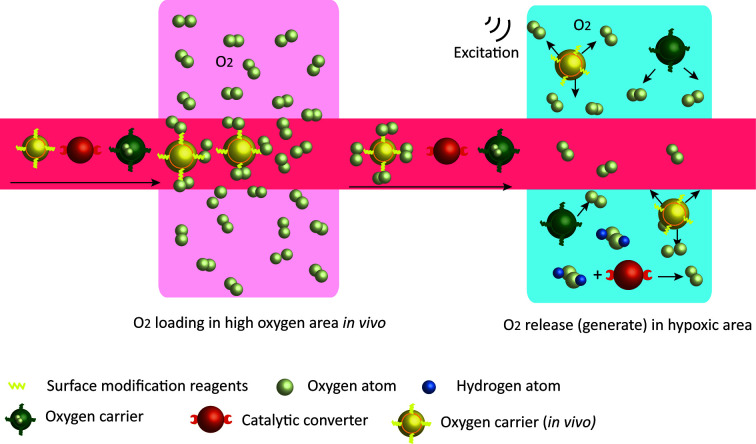
Strategies of nanoparticles to increase tissue oxygen content. Oxygen carriers wrap O2 *in vitro*, or bind O2 in high oxygen areas *in vivo* and release them in low oxygen environment. Nanoparticles with catalytic effects react with excessive endogenous H2O2 in the TME to generate oxygen.

Oxygen-carrying is a direct strategy in which nanocarriers load oxygen in oxygen-rich areas and release oxygen in hypoxic areas depending on the partial oxygen pressure (**Figure 2**) (53). Perfluorocarbon is a safe O_2_ carrier that has been already demonstrated in clinic, and the encapsulation of perfluorocarbon with albumin enhanced its accumulation in the tumor site and rapidly released the oxygen that was physically dissolved (69). Fluorocarbon-functionalized nanoparticles enhanced the effects of both photodynamic therapy (PDT) (70) and oxygen-sensitive anti-tumor drugs (71) by increasing tumor oxygenation. Besides, perfluorocarbons have entered clinical trials for ischemia and imaging theranostic strategies to ensure that the simple O_2_ transport system can be rapidly and easily transformed into clinical applications. Hemoglobin (Hb) is another appreciating functional material for the development of oxygen-carrying nanoparticles. Hemoglobin nanoparticles (H-NPs) are assembled after re-emulsion. They are Hb-based oxygen nanocarriers that attenuate the hypoxia-induced decrease in decitabine activity and sensitize renal cell carcinoma to combination therapy of decitabine with oxaliplatin (72). Overall, hypoxic TME is a critical variable for immunotherapy. The development of nanomaterials targeting the hypoxic TME is one of the fastest growing branches of nanomedicine.

### Nano-Based Photothermal Therapy Induced Tumor Immune Response

By effectively generating lethal doses of heat under near-infrared (NIR) light irradiation, photothermal therapy adopts material with high photothermal conversion efficiency to kill tumor cells (73, 74). The nanomaterials that initially provided photothermal therapy were mainly precious metals, but they have gradually developed into nanocarbons, metal organic compounds and organic dyes. For instance, PLGA nanoparticles loaded with indocyanine green (ICG) stimulate physicochemical and physiological changes in TME under mild heating, leading to increased infiltration of chondroitin sulfate proteoglycan-4 (CSPG4)-specific CAR T cells (73). Silica sealed by gold nanoshells (AuroShell) is the only inorganic material approved by Food and Drug Administration (FDA) for clinical photothermal therapy (75). AuroShell particles can be passively accumulated in solid tumors through the vasculature and were demonstrated safe when they were used systemically in focal ablations in prostate (74).

**Figure 3:**
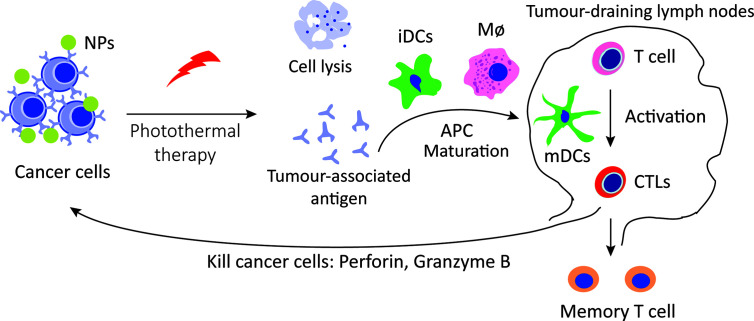
Immunotherapy induced by photothermal therapy. Photothermal therapy increases the tissue immunogenic cell death and release antigens, which are presented to T cells by DCs and macrophages, enhance the recognition and killing to tumor cells.

Intriguingly, tumor immune effect induced by photothermal therapy has been recognized. Photothermal therapy induces deep tissue immunogenic cell death, potentiates cancer immunotherapy and synergistically enhances immune efficacy (**Figure 3**). Gold nanostars (GNS) induced the anti-tumor immune response following the highly immunogenic thermal death of cancer cells, and the combination of GNS-mediated photothermal therapy with ICB reversed tumor-mediated immunosuppression (76). Al_2_O_3_ nanoparticle coating with polydopamine acts as an adjuvant for photothermal therapy, triggering a series of powerful cell-mediated immune responses to eliminate residual tumor cells and reduce the risk of tumor recurrence (77).

The therapeutic outcome of photothermal therapy is limited by the degree of light transmission (78), while the deep internal area of the tumor lacks lymphocytic infiltration and experiences in various immune escape mechanisms (3). However, these issues could be solved by the combined nano-based photothermal therapy with immunotherapy. A multiplex nanoparticle assembled by a NIR photosensitizer named IR780 and an IDO inhibitor named NLG919 enhanced accumulation in the tumor site *via* passive targeting, increased the infiltration and differentiation of T cells into CD8^+^ T cells, suppressed the tumor margin beyond the border of effective photothermal therapy and strengthened the immune response to inhibit the distal tumor (78).The assembly of gold nanoparticles into fluid liposomes produced photothermal sensors that have NIR-I and NIR-II biological windows and respond to different absorptions of red light. NIR-II light activates both innate and adaptive immune responses, achieves effective tumor control (3) and triggers more homogeneous and deeper immunogenic cancer cell death than NIR-I light. In addition, photothermal therapy facilitates the accumulative and effective function of CAR-T cells within solid tumors by reducing tumor interstitial pressure, increasing blood perfusion and releasing antigens (73). Briefly, these findings demonstrate the great potential of nano-based photothermal therapy in immunotherapy.

### Reprogramming the Immune Microenvironment

The efficacy of immunotherapy depends on the infiltration of immune cells and immune factors in TME. Tumor cells reprogram the microenvironment to facilitate immune escape and induce immunotherapy tolerance. Therefore, reversing the immunosuppressive microenvironment is an insightful perspective for the improvement of immunotherapy. The increasing numbers of multifunctional modified nanoparticles are being created, which shows us a diversity of anti-tumor mechanisms. In addition to a role of carrier, nanoparticles also directly activate immune cells to participate in anti-tumor responses.

At present, most of the nanoparticles that activate immune cells are coupled with immune activators, such as ICB molecules (34, 79, 80) and tumor vaccines. Luo and colleagues first discovered that Fe_3_O_4_ NPs act as immunopotentiators to stimulate dendritic cell (DC)-based immunotherapy and to potentially activate macrophages and T cells. Fe_3_O_4_-OVA vaccines successfully inhibit the subcutaneous growth and lung metastasis of melanoma (1). Because iron-based nanomaterials have been approved by FDA, they are expected to be used in clinical tumor treatment in future. Nanospheres, as a new vaccine adjuvant, elicited prominent antigen cross-presentation effects on DCs and bone marrow dendritic cells (BMDCs), enhanced humoral and cellular immune responses *in vivo* (81, 82). Although the application of ICB is impeded by TME, the modified nanoparticles co-loaded with CRISPR/Cas9 and paclitaxel (PTX) to reduce Tregs and repolarize tumor associated macrophages (TAMs), which can reverse the TME and enhance anti-tumor immunity (83). Collectively, we can continuously obtain the optimal efficacy of immunotherapy thanks to the nanomaterials’ function of crosstalk reprogramming and immune cells activation in various ways.

Interestingly, some nondrug-loaded nanoparticles have been shown to directly modulate the immune microenvironment. Zhang et al. assembled ursonic acid with liposomes into nanoparticles to increase the solubility, which modulated TME by reducing CD4^+^CD25^+^Foxp3^+^ Tregs, and this reduction was correlated with the inhibition of STAT5 phosphorylation and the reduction of IL-10 expression (81). Systemic exposure to nanoparticles enabled transient immune recognition of tumor, increased the number of immune cells, such as NK cells, monocytes, CD4^+^ T cells, and CD8^+^ T cells, reconfigured TME immune system and delayed tumor growth; Of note, all of these changes were independent of antibody therapeutic activity and therapeutic payload (84). Natural nanoparticles can be easily obtained with their application prospects. Nanoparticles extracted from cuttlefish ink (CINPs) increase CD8^+^ T cells and repolarize M2 macrophages to the M1 phenotype through activation of the MAPK pathway. CINPs almost completely restrained tumor growth when synergizing with the photothermal effect, which induces tumor-specific antigen release (85). In general, the immunosuppressive microenvironment of solid tumors represents a severe obstacle for immunotherapy. However, nanomedicine potentiates the effects of TME modulation by activating immunosupportive cells and inhibiting immunosuppressive cells.

In addition to tumor tissues, LN is another important target for nanoparticles to produce anti-tumor effects. For instance, compared with its parent compound, the structure-optimized CpG-DNA/peptide vaccine increases the efficiency of T cell initialization and improves anti-tumor function and reduces systemic toxicity due to its advantages of a significant increase in LN accumulation and a decrease in systemic diffusion (86). Nanoparticle-bound tumor-associated antigens can transform immunosuppressive environment of the LNs draining the tumor into the more immunogenic environment. Compared with non-targeted vaccines, vaccines that target tumor draining lymph nodes (tdLN) can regress tumors and retain a higher host survival rate, because they induce intense cytotoxic CD8 ^+^ T cells reactions (87).

Nanomaterials increase anti-tumor effects in the progress of both cell-mediated immunity and humoral immune regulation. For one thing, multiple nanoparticles effectively stimulate the proliferation of CD4^+^ and CD8^+^ T cells, thereby further promote the production of antibodies, which together trigger more dramatic humoral and cellular immune responses than free antigen alone (88). For another, some nanoparticles can directly increase the binding robustness of antibodies. As a result, manipulating the chemistry of polyanhydride nanoparticles promotes the differential kinetics of antibody titer, affinity and epitope specificity development, and eventually, induces continuous and mature antibody responses (89).

### Nano-Engineered Cells

**Figure 4:**
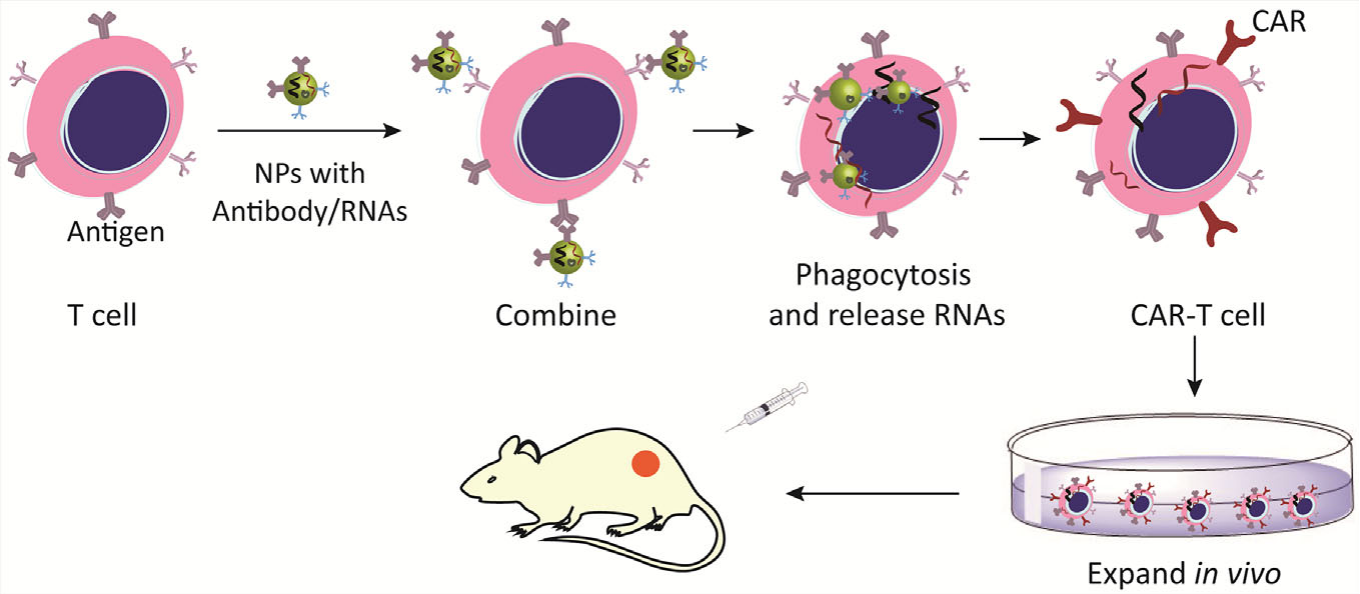
Nanomaterials activate and expand adoptive T cells. Nano-modified CAR-T cells increase the expression of CAR antigens and improve the recognition of tumor cells.

Currently, viral delivery vector is a major medium of chimeric antigen receptor (CAR)-engineered T cells, which induce permanent expression of CAR but may cause serve adverse reactions (90). Nanotechnology promotes the adoptive T cell therapy, effectively delivers CAR cargo to T cells or other effector cells, and then quickly programs T cells to recognize tumor cell antigens (91). Nanocarriers present TCR stimulation signals or pro-survival cytokines, accelerate T cell transduction *in vivo* and improve their survival rate (91). Biodegradable nanomaterials take advantages of structural flexibility, the ability to deliver soluble paracrine to T cells and surface-bound molecules will be lost over time, which prevents excessive activation of T cells (92). In a word, nanotechnology-based CAR T cell modification is a promising approach to improve the efficacy of adoptive cell therapy (**Figure 4**).

Nanotechnology efficiently delivers pEGFRvIII-CARs to Jurkat T cells transiently and expresses EGFRvIII-CAR on the transfected cell membrane, enabling Jurkat T cells specifically to recognize and bind to EGFRvIII-positive tumor cells (93). Nanoparticles which encapsulated with an A2aR-specific small molecule antagonist attached to the surface of CAR-T cells *in vitro* had no effect on the recognition of target cells, IFN-γ secretion, cell cytotoxicity or migration but increased the active targeting of the tissue of interest and ameliorated intratumoral T cell hypofunction (94).

Therapeutic nanoparticles click into the surface of CAR-T cells modified with IL-13 targeting quadruple mutant (TQM-13), which increases the affinity with glioblastoma (95). Nanoparticles encoding FOXO1 gene promote the transformation of effector T cells into memory cells (96). Compared with traditional methods, ionizable lipid nanoparticles deliver mRNA to T cells to induce CAR expression in an equal level, but decrease cytotoxicity and enhance the potential of mRNA-based CAR-T cell engineering methods (90). Conjugating IL-2 to liposomes is inclined to target adoptive cell therapy (ACT) cells and induce ACT-T cell proliferation in tumor-tolerant mice, proving the feasibility of repetitive functional targeting of T cells *in vivo* (97). Dextran-coated superparamagnetic iron oxide nanoparticles have been designed for the expansion of T cells, while MHC-Ig antibodies and anti-CD28 antibodies are conjugated to them to provide antigen-specific and co-stimulatory signals. This Nano-aAPC provoked the activation of tumor-specific T cells through “enrichment plus amplification” (98).

In addition, nanoparticles can self-assemble into CAR-like complex which activate T cells while targeting tumors. Bai et al. developed CAR-like multivalent aptamer nanoparticles which were assembled with CD28 RNA aptamer and the tetramer of CTLA-4 RNA aptamer, as well as a folic acid labeled single stranded DNA fragment in a stable nucleic acid three-way junction scaffold. These nanoparticles increase T cell proliferation, reverse the inhibitory effect of IL-2 secreting caused by exogenous B7.1 molecules on T cells *in vitro* and promise a novel approach to develop a multi-functional design of aptamer drugs with potential CAR-like characteristics to enhance the safety of CAR-T cell immunotherapy (99).

Nanoparticles also harbor both the function of NK cells modification (100) and the potential of CAR-NK therapy achievement. For one thing, nanoparticles promote the transfection of siRNA to NK92 cells which have been used for NK-based cancer immune therapy in clinical trials (101); For another, nanoparticles are used to enhance the expansion of adoptively transferred NK cells. For instance, particles prepared from the plasma membrane of K562-mb21-41BBL cells that express 562BBL and membrane-bound IL-21 (PM21 particles) induce PBMCs from healthy donors and patients with AML to produce specific NK cell expansion (102).

### Combination of Nanotechnology With Monoclonal Antibodies Therapy

The combination of Abs with nanomedicine includes nanobiosensors, Ab-based nanomachines and active targeted drug delivery systems (103). Some tumor-specific markers have been discovered, and the usage of corresponding Abs modified nanoparticles can transform the passive targeting into active condition and functionalize them as “guided missiles” to increase the specificity of enrichment in tumor sites and to minimize damage to healthy tissues. For example, anti-CD133 Ab-conjugated SN-38-loaded nanoparticles recognize CD133 on the surface of colorectal cancer cells, thereby increasing the targeting of nanometers (104). CD-340-conjugated DOX-loaded PLGA nanoparticles preferentially deliver drug to breast cancer tissue, and reduce DOX-mediated cardiotoxicity due to its tumor-specific distribution (105). In addition, antibodies for more markers were conjugated to a variety of nanoparticles which have been extensively verified in preclinical studies. Most of them have shown good targeting and anti-tumor activity. For example, daunorubicin-loaded CdTe QDs conjugated to anti-CD123 mAbs (DNR-CdTe-CD123) treatment was designed for high-risk myelodysplastic syndromes (MDS) (106), the nanocomposite displayed higher inhibition rate and apoptosis rate in MDS cells than monotherapy, enhanced the therapeutic efficacy and reduced the side effects of daunorubicin. Genetically engineering cell-derived exosomes with anti-CD3 and anti-HER2 antibodies (aCD3-aHER2 SMART-Exos) dually target T cells and HER2^+^ breast cancer, selectively inducing HER2-expressing tumor-specific immunity and activating cytotoxic T cells toward attacking breast cancer cells (107).

At present, some clinically approved drugs are gradually being nano-sized to improve their therapeutic effects. Herceptin-conjugated paclitaxel loaded PCL-PEG worm-like nanocrystal micelles (PTX@PCL-PEG-Herceptin) remained relatively stable in the circulation and in TME. PTX@PCL-PEG-Herceptin greatly enhanced the binding robustness of the nanoparticle to the HER2^+^ breast cancer cells, enriched target cells rapidly and protected normal tissues from the toxic effects (108). The conjugation of HER2 protein 1-146 with cholesteryl pullulan (CHP) nanoparticles (also named CHP-HER2 vaccine) was safer than HER2 protein 1-146 used only, the complex induced HER2-specific CD8^+^ and CD4^+^ T cell immune responses in patients who received four to eight vaccinations (109).

Anti-EGFR Abs are mainly used to bind gold nanoparticles based on active targeting function (110, 111), and phase I clinical trials have been launched on the basis of a large number of preclinical studies. For example, anti-EGFR-immunoliposomes loaded with doxorubicin (C225-ILs-dox) were used for patients with relapsed or refractory high-grade gliomas (NCT03603379). Anti-EGFR ILs-dox nanoparticles were designed by pegylated liposomes which encapsulated doxorubicin into antigen-binding fragments (Fab’) of cetuximab, this nanoplatform was well tolerated, and showed more effective than non-targeted liposomes at destroying EGFR-overexpressing target cells because of its active and specific internalization (NCT01702129) (112).

## Possibilities of Nanomaterials in Clinical Tumor Immunotherapy

A large number of nanomaterials have been synthesized for tumor treatment in clinical trials (**Supplementary Table S3**). However, there are few approved in clinical applications. Only liposomes and albumin carriers were formed into stable dosage types. The clinical nanomaterials applied for tumor therapy are listed in **Supplementary Table S4**.

Liposome is an outstanding drug delivery platform due to its biocompatibility and drug encapsulating efficiency (100), which can be loaded with hydrophobic and hydrophilic molecules. Immunoliposomes directly target tumors to provide a bystander killing effect through diffusion of loading agents to neighboring tumor cells (42, 112). Liposomes have been extensively applied in clinical management of cancers. The first nanomedicine for tumor clinical treatment is doxorubicin (Doxil™), a PEGylated long-circulating liposome loaded with doxorubicin approved in 1995, whose main indications are advanced ovarian cancer, multiple myeloma and HIV-combined Kaposi’s sarcoma (113). Nano-platform based vincristine sulfate liposome (Marqibo™) was proved for treatment of adult patients with Philadelphia chromosome–negative (Ph^-^) acute lymphoblastic leukemia (ALL) (114, 115) while Irinotecan liposome (Onivyde™) for metastatic pancreatic cancer (116). Other liposome-based nano-drug in tumor treatment includes daunorubicin liposome (DaunoXome^®^), doxorubicin liposomes, cisplatin liposomes and so on. Furthermore, modification of nano-liposomes increases the functional characteristics of the nano-drug platform with greater targeting effect. Temperature-sensitive liposomes could passively load with gemcitabine and copper complex to release drugs with tumor vasculature in response of ultrasound hyperthermia (117). Oleuropein loaded folate-targeted-PEG liposomes was prepared by the method of film hydration-cum-extrusion technique which enhanced the anti-cancer effect of oleuropein (118).

Albumin is a well-tolerated material with the advantages of highly solution and stability, no toxicity, no immunogenicity and easily chemical function (119). Albumin could protect circulating nanoparticles from the recognition and elimination of mononuclear phagocytic systems with half-life of 19 days, and they can avoid renal clearance because of the reabsorption by receptor-mediated endocytosis in the renal proximal tubule (120), thus albumin-based nanoparticles can be enriched in solid tumor tissues based on their increased consumption by cancer cells and the interaction with TME (121). All of these processes make nanoparticles become helpful carriers for anti-cancer agents. Onafuye and colleagues designed doxorubicin-loaded albumin nanoparticles by desolvation and crosslink using glutaraldehyde which could reverse transporter mediated drug resistance, whereas other nano-carrier systems have not found the similar effect (122). Choi and colleagues prepared inhalable self-assembling doxorubicin albumin nano-system which was treated with tumor necrosis factor (TNF)-related apoptosis-inducing ligand (TRAIL) and was based on albumin, demonstrating synergistic anti-tumor efficacy, and providing a new inhalation-based combination therapies to treat drug-resistant lung cancer with the obvious reduction of the dose of doxorubicin (119). Currently, the most extensively used albumin nanoparticles is nab-paclitaxel (Abraxane™), which approved for the treatment of breast cancer, metastatic non-small cell lung cancer (NSCLC) and pancreatic cancer. Besides, there are still many clinical trials based on the combination of nab-paclitaxel and other drugs (such as cisplatin, oxaliplatin, toripalimab, and S1) to obtain better therapeutic effect through combination therapy.

Polyethylene glycol (PEG) is the chief ingredient used to synthesize nanoparticles and modify the surface of nanomaterials. PEG-b-PLA micelles are the first-generation platform with a hydrodynamic diameter of 33 nm in the usage of systemic multiple administration of poorly water-soluble anti-cancer agents (123). The combination of PEG-b-PLA micelles with paclitaxel (Genexol-PM^®^) has been used in clinical cancer treatment with a high response rate in clinical trials in patients with NSCLC, gastric cancer, breast cancer and fewer acute hypersensitivity reactions (123).

Cyclodextrins (CDs) and other polymers were often combined with water-insoluble pharmaceutical drugs to increase their solubility and availability (124). Cyclodextrins- camptothecin (CRLX101) complex optimized the plasma pharmacokinetics and then facilitated drug delivery to tumors (125). The preferential uptake of cyclodextrins and camptothecin conjugation may promote the selective release of tumor antigens into TME and enhance the effect of tumor immune drugs (126). Preclinical studies have proven that CRLX101 could reduce adverse reactions and increase NK cell and T cell populations, which may potentially improve the anti-PD1/PDL1 therapy (127).

Cholesteryl pullulan (CHP) nanogel is a novel antigen delivery system based on CHP nanogel that has been accomplished phase I clinical trials. CHP is conjugated to HER2 and NY-ESO-1 antigen-related cancer vaccine. Additionally, HP-NY-ESO-1 is a safe and promising cancer vaccine. In previous studies, CHP-NY-ESO-1 vaccination showed CD4^+^ and CD8^+^ T cell response activities (128). The addition of anti-PD-1 activates NY-ESO-1 specific T cells as well as other tumor antigen-reactive T cells. Afterwards, clinical trials of combined therapies including cancer vaccines, adjuvants and ICBs, are ready to proceed (129). Comparatively, the CHP-HER2 vaccine is well tolerated, and HER2-specific CD8^+^ and CD4^+^ T cell immune responses have been detected in patients who had received the vaccine (109).

Autophagy in tumor cells also plays a key role in the cross-presentation of tumor antigens (130). Tumor-derived autophagasomes vaccine named DRibbles is defined as a novel type of multivalent vaccine which was produced by disrupting the ubiquitin proteasome system to degrade intracellular proteins. DRibbles vaccine consists of autophagic vesicles which are rich in defective ribosomal products and short-lived proteins, known tumor-associated antigens, mediators of innate immunity and surface markers that promote phagocytosis and cross-presentation of antigen-presenting cells (131). Human antigen-specific memory T cells can be activated by specific viral antigens during immune monitoring and adoptive immunotherapy. As a result, Dribbles vaccine will be a vaccine for cancer patients in the future (132). In the phase I of clinical trials, DRibbles had been derived from autologous tumor cells and applied to vaccinate patients with NSCLC, following a randomized multi-center phase II clinical trial was initiated to use allogeneic DRibbles vaccine (132). The available evidence supported that DRibbles vaccine is a human treatment strategy in the latest clinical development and it promises to be a delivery mechanism for other vaccines in the future.

Virus-like particles (VLPs) are nanoparticles that self-assembled by one or more viral proteins, with the diameter of about 10-200nm (133). VLPs do not contain nuclei acids which exhibit non-infectious function for the vaccinated individuals. By contrast, the structure of VLPs are similar to the conformation of wild type viruses which can activate adaptive immunity (134). VLPs can be modified by chemical or genetic fusion technologies to express chimeras or targeted delivery of small molecule drugs and nucleic acids as a result of the improvement of bioavailability of the delivered substance (135). Thus, VLPs can restore immune response, cross-present and induce CTL responses. Furthermore, VLPs identify and combine specific pattern recognition receptors on the surface of DCs followed by internalizing into activate DCs, and then present peptides which was loaded into MHC-I/II. Eventually, they in turn initiate CD8^+^ or CD4^+^ T cell immunity (134). Collectively, the ability to engineer VLPs with exquisite detail makes them popular candidates for the design of a platform to produce vaccines against various diseases.

## Application Prospects of Nanomedicine in Immunotherapy

**Figure 5:**
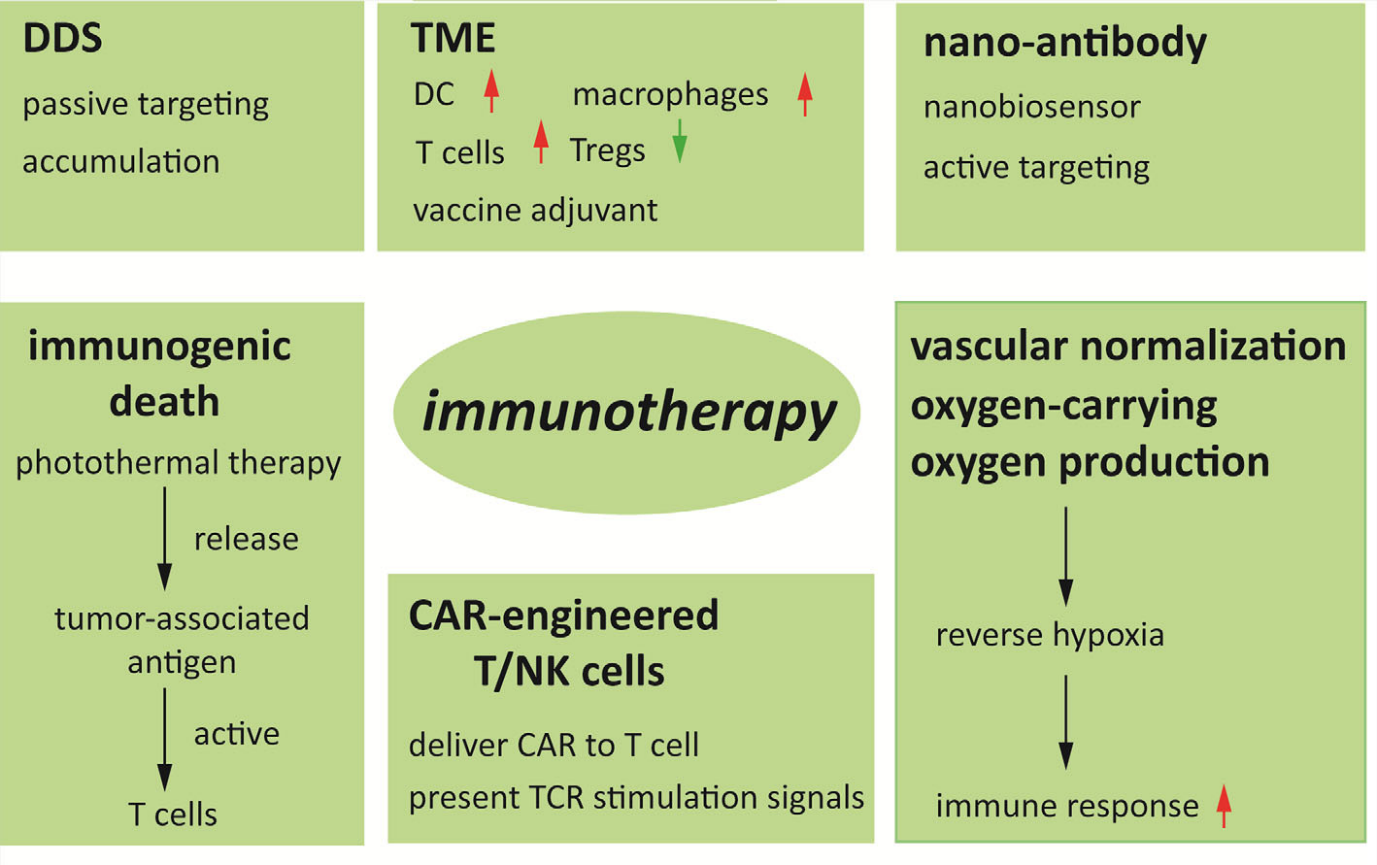
Mechanism of nanoparticles in immunotherapy.

Immunotherapy has become a vital tool of cancer treatment. The effectiveness of immunotherapy is various in tumor subtypes. The development mechanism of primary tumors and immune regulation mechanisms have been the key to clarify the difference in efficacy of immunotherapy. Even so, the hurdles existed in conventional tumor immunotherapy hindered the progress of tumor immunotherapy. With the advancement of nanotechnology, the use of nanomaterials should not be ignored in tumor therapy due to their intrinsic immune modulation activities. The auxiliary applications of nanomaterials will provide a predictable guarantee for the effectiveness and safety of immune interventions (**Figure 5**). Nanomedicines take advantages of immunotherapies in four aspects. 1) Immunomodulators, such as immunopharmaceuticals, vaccines, siRNAs, etc. carried by nanomaterials can be slowly released on specific targets to prolong the effect of immunotherapy and reduce systemic side effects. 2) The photothermal effect mediated by nanomaterials triggers immunogenic cell death. 3) The modified nanomaterials can activate cytotoxic T cells and antigen-presenting cells, reverse the polarization of macrophages and inhibit Treg cells, thereby enhancing the tumor killing effect. 4) Regulating tumor blood vessels and tumor hypoxic microenvironment not only enhances the sensitivity of immunotherapy, but also strengthens the therapeutic effect of radiotherapy and chemotherapy. In summary, nanomedicine has extremely high variability in material and particle size selection, surface modification, packaged drug selection, and drug delivery schemes. Therefore, there is extensive optimization space for nanomedicine to improve. In the past decades, the practicality of biomaterials has been verified in the field of tumor therapy. Afterwards, more than ten kinds of nano-based drugs have been approved for tumor or other diseases clinical treatment. And now, the clinical transformation of material-based cancer immunotherapy is accelerating. In the future, the principles we have learned from the existing experience of using nanomaterials will guide us to design more effective cancer immunotherapies, allowing for extend the frontiers of more successful cancer treatment.

## Author Contributions

WL wrote the majority of the manuscript. AP cowrote the manuscript. HW and YQ drew images and summarized tables. YL supervised the research. LL and MC designed and cowrote the manuscript, and supervised the research. All authors contributed to the article and approved the submitted version.

## Funding

This work is partially supported by National Key Research and Development Program of China (No. 2017YFA0205200) and the National Natural Science Foundation of China (No. 81571785, 81771957, 81801811).

## Conflict of Interest

The authors declare that the research was conducted in the absence of any commercial or financial relationships that could be construed as a potential conflict of interest.
