# Supplementary material for: Anti-Cancer Nanomedicines: A Revolution of Tumor Immunotherapy
Source: Front Immunol. 2020 Dec 21;11:601497. doi: 10.3389/fimmu.2020.601497 (PMC7779686; doi:10.3389/fimmu.2020.601497)
Supplement: Supplementary file 1 [file DataSheet_1.zip › Supplementary Table 1.DOCX]

Supplementary Table S1 Specific indicators of cancer immunotherapy in clinical trials

| Molecular | Agent | Disease | Combination | Phase | Start  Completion | Status | Identifier |
| --- | --- | --- | --- | --- | --- | --- | --- |
| LAG-3 | Sym022 | Advanced solid tumor malignancies or lymphomas | NA | I | 2018  2020 | Completed | NCT03489369 |
|  |  |  | Sym021, Sym023 | I | 2017  2021 | Recruiting | NCT03311412 |
|  | BMS-986016  (Relatlimab) | Neoplasms by site | Nivolumab  BMS-986213 | I | 2013  2023 | Recruiting | NCT01968109 |
|  |  | Hematologic neoplasms | BMS-936558 | I | 2014  2020 | Active, not recruiting | NCT02061761 |
|  |  | Cancers | Nivolumab | I | 2016  2020 | Recruiting | NCT02966548 |
|  |  | Refractory MSI- H solid tumors | Nivolumab | II | 2018  2022 | Recruiting | NCT03607890 |
|  |  | Relapsed refractory multiple myeloma | Elotuzumab  Pomalidomide Dexamethasone | I/II | 2020  2022 | Recruiting | NCT04150965 |
|  |  | Various advanced cancer | Nivolumab  Ipilimumab  Daratumumab | I/II | 2015  2022 | Active, not recruiting | NCT02488759 |
|  |  | Colorectal adenocarcinomas | Nivolumab | II | 2018  2023 | Recruiting | NCT03642067 |
|  |  | Advanced cancer | Nivolumab  BMS-986205  Ipilimumab | I/II | 2018  2022 | Recruiting | NCT03459222 |
|  |  | Advanced cancer | Nivolumab  Dasatinib  Ipilimumab  BMS-986205 | II | 2016  2021 | Active, not recruiting | NCT02750514 |
|  |  | Microsatellite stable /unstable colorectal cancer  Mismatch repair deficient / proficient colorectal cancer | Ipilimumab  Nivolumab  Cobimetinib  Daratumumab | II | 2014  2022 | Active, not recruiting | NCT02060188 |
|  |  | Advanced gastric cancer | Nivolumab  Ipilimumab  BMS-986205  Rucaparib | II | 2016  2022 | Recruiting | NCT02935634 |
|  |  | Gliosarcoma  Recurrent brain neoplasm | Anti-PD-1  Anti-CD137 | I | 2016  2022 | Recruiting | NCT02658981 |
|  |  | Advanced cancer | Nivolumab  Ipilimumab  BMS-986205  BMS-813160 | II | 2017  2022 | Recruiting | NCT02996110 |
|  |  | Gastric cancer  Esophageal cancer  GastroEsophageal cancer | Nivolumab  Carboplatin  Paclitaxel  Radiation | I | 2017  2023 | Recruiting | NCT03044613 |
|  |  | Head and neck squamous cell carcinoma | Nivolumab  Ipilimumab | II | 2019  2024 | Recruiting | NCT04080804 |
|  |  | Advanced cancer | Nivolumab  Cabiralizumab  Ipilimumab  IDO1 Inhibitor  Radiation Therapy | I | 2018  2022 | Recruiting | NCT03335540 |
|  |  | Chordoma | Nivolumab | II | 2019  2022 | Recruiting | NCT03623854 |
|  |  | Glioblastoma | Nivolumab | I | 2018  2021 | Recruiting | NCT03493932 |
|  |  | Gastric cancer  Adenocarcinoma of the esophagogastric junction | Nivolumab  Ipilimumab  Oxaliplatin  Docetaxel  5-Fluorouracil  Folic acid | II | 2019  2025 | Recruiting | NCT04062656 |
|  |  | Melanoma | Ipilimumab  Nivolumab  Conventional Surgery | II | 2016  2020 | Recruiting | NCT02519322 |
|  |  | Melanoma | Nivolumab | II | 2019  2022 | Recruiting | NCT03743766 |
|  |  | Squamous cell carcinoma of the head and neck | Nivolumab+Ipilimumab | II | 2020  2023 | Not yet recruiting | NCT04326257 |
|  | TSR-033 | Advanced solid tumors  Colorectal cancer | Anti-PD-1 | I | 2017  2021 | Recruiting | NCT03250832 |
|  |  | Advanced or metastatic solid tumors | TSR-022  DTSR-042 | I | 2016  2022 | Recruiting | NCT02817633 |
|  | IMP321  (Eftilagimod alpha) | Solid tumors  Peritoneal carcinomatosis | Avelumab | I | 2017  2020 | Recruiting | NCT03252938 |
|  |  | Metastatic breast cancer | NA | I | 2006  2010 | Completed | NCT00349934 (1) |
|  |  | Metastatic breast cancer | Paclitaxel | I | 2020  2022 | Not yet recruiting | NCT04252768 |
|  |  | Adenocarcinoma breast stage IV | Paclitaxel | II | 2015  2020 | Active, not recruiting | NCT02614833 |
|  |  | Non-small cell lung cancer  Head and neck squamous cell cancer | Pembrolizumab | II | 2019  2021 | Recruiting | NCT03625323 |
|  |  | Stage IV melanoma  Stage III melanoma | Pembrolizumab | I | 2016  2019 | Completed | NCT02676869 |
|  |  | Stage IV renal cell carcinoma | NA | I | 2005  2008 | Completed | NCT00351949 (2) |
|  |  | Melanoma (Skin) | Melan-A VLP vaccine,  cyclophosphamide  fludarabine phosphate | I | 2005  2011 | Completed | NCT00324623 (3) |
|  | GSK2831781 | Healthy volunteers | NA | I | 2019  2019 | Completed | NCT03965533 |
|  |  | Colitis, Ulcerative | NA | II | 2019  2023 | Recruiting | NCT03893565 |
|  |  | Psoriasis | NA | I | 2014  2018 | Completed | NCT02195349 |
|  | REGN3767 | Malignancies | Cemiplimab | I | 2016  2021 | Recruiting | NCT03005782 |
|  |  | Breast tumors  Angiosarcoma | Cemiplimab | II | 2010  2026 | Recruiting | NCT01042379 |
|  | RO7247669  (+PD-1) | Solid tumors | NA | I | 2019  2022 | Recruiting | NCT04140500 |
|  | MGD013  (+PD-1) | Gastric cancer | Niraparib | I | 2020  2022 | Recruiting | NCT04178460 |
|  |  | Gastric cancer  Gastroesophageal junction cancer  HER2-positive gastric cancer | Margetuximab  Chemotherapy | II/III | 2019  2026 | Recruiting | NCT04082364 |
|  |  | Advanced solid tumors  Hematologic neoplasms | Margetuximab | I | 2017  2022 | Recruiting | NCT03219268 |
|  |  | Advanced hepatocellular carcinoma | Brivanib Alaninate | I/II | 2020  2023 | Recruiting | NCT04212221 |
|  | FS118(+PD-1) | Advanced cancer  Metastatic cancer | NA | I | 2018  2020 | Active, not recruiting | NCT03440437 |
|  | LAG525 | Advanced solid tumors | PDR001 | I/II | 2015  2020 | Active, not recruiting | NCT02460224 |
|  |  | Triple-negative breast cancer | Spartalizumab  Carboplatin | II | 2018  2021 | Active, not recruiting | NCT03499899 |
|  |  | Triple negative breast cancer | Spartalizumab  NIR178  Capmatinib  MCS110  Canakinumab | I | 2019  2022 | Recruiting | NCT03742349 |
|  |  | Advanced solid and hematologic malignancies | PDR001 | II | 2018  2020 | Active, not recruiting | NCT03365791 |
|  |  | Melanoma | Spartalizumab  Capmatinib  Canakinumab  Ribociclib | II | 2018  2021 | Recruiting | NCT03484923 |
|  | INCAGN02385 | Advanced malignancies | NA | I | 2018  2020 | Recruiting | NCT03538028 |
|  | XmAb®22841  (+CTLA4) | Advanced solid tumors | Pembrolizumab | I | 2019  2027 | Recruiting | NCT03849469 |
| TIM-3 | Sym023 | Metastatic cancer  Solid tumor  Lymphoma | NA | I | 2018  2020 | Active, not recruiting | NCT03489343 |
|  |  | Metastatic cancer  Solid tumor  Lymphoma | Sym021  Sym022 | I | 2017  2021 | Recruiting | NCT03311412 |
|  | TSR-022 | Advanced or metastatic solid tumors | SR-042  TSR-033 | I | 2016  2022 | Recruiting | NCT02817633 |
|  |  | Adult primary liver cancer | TSR-042 | II | 2019  2023 | Recruiting | NCT03680508 |
|  |  | Solid tumors | TSR-042 | I | 2017  2021 | Active, not recruiting | NCT03307785 |
|  |  | Melanoma stage III  Melanoma stage IV | Dostarlimab | II | 2020  2024 | Recruiting | NCT04139902 |
|  | RO7121661  (+PD-1) | Solid tumors | NA | I | 2018  2022 | Recruiting | NCT03708328 |
|  | LY3321367 | Solid tumors | LY3300054 | I | 2017  2020 | Active, not recruiting | NCT03099109 |
|  |  | Solid tumors | LY3300054 | I | 2016  2021 | Recruiting | NCT02791334 |
|  | BGB-A425 | Locally advanced or metastatic solid tumors | tislelizumab | I/II | 2018  2021 | Recruiting | NCT03744468 |
|  | MBG453 | Advanced malignancies | PDR001  Decitabine | I/II | 2015  2021 | Recruiting | NCT02608268 |
|  |  | Acute myeloid leukemia | Venetoclax  Azacitidine | II | 2020  2025 | Not yet recruiting | NCT04150029 |
|  |  | Leukemia, Myeloid,  Acute myelodysplastic syndromes  Preleukemia  Bone marrow diseases  Hematologic diseases | Decitabine  PDR001 | I | 2010  2021 | Recruiting | NCT03066648 |
|  |  | Primary myelofibrosis  Myelofibrosis  Post-essential thrombocythemia myelofibrosis  Post-polycythemia vera myelofibrosis | NIS793  Spartalizumab  Decitabine | I | 2020  2023 | Not yet recruiting | NCT04283526 |
|  |  | Glioblastoma multiforme | NA | I | 2020  2024 | Active, not recruiting | NCT03961971 |
|  |  | Myelodysplastic syndromes  Leukemia, Myelomonocytic, Chronic | Azacitidine | III | 2020  2027 | Not yet recruiting | NCT04266301 |
|  |  | Acute myeloid leukemia (AML)  High-risk myelodysplastic syndrome (MDS) | HDM201  Venetoclax | I | 2019  2021 | Recruiting | NCT03940352 |
|  |  | Myelodysplastic syndromes | Hypomethylating agents | II | 2019  2023 | Recruiting | NCT03946670 |
|  | INCAGN02390 | Advanced malignancies | NA | I | 2018  2021 | Recruiting | NCT03652077 |
| TIGIT | BMS-986207 | Solid tumors | Nivolumab | I/II | 2016  2022 | Recruiting | NCT02913313 |
|  |  | Relapsed refractory multiple myeloma | Elotuzumab, Pomalidomide, Dexamethasone | I/II | 2020  2022 | Recruiting | NCT04150965 |
|  | BGB-A1217 | Metastatic solid tumors | Tislelizumab | I | 2019  2021 | Recruiting | NCT04047862 |
|  | MTIG7192A | Advanced/metastatic tumors | Atezolizumab | I | 2016  2022 | Recruiting | NCT02794571 |
|  |  | Non-small cell lung cancer | Atezolizumab | II | 2018  2020 | Active, not recruiting | NCT03563716 |
|  |  | Small cell lung cancer | Atezolizumab  Carboplatin  Etoposide | III | 2020  2023 | Recruiting | NCT04256421 |
|  |  | Non-small cell lung cancer | Atezolizumab | III | 2020  2025 | Recruiting | NCT04294810 |
|  | AB154 | Solid tumors | AB122 | I | 2018  2020 | Recruiting | NCT03628677 |
|  |  | Non small cell lung cancer  Squamous /nonsquamous non small cell lung cancer | Zimberelimab  AB928 | II | 2020  2022 | Recruiting | NCT04262856 |
|  | IBI939 | Advanced malignancies | Sintilimab | I | 2020  2023 | Not yet recruiting | NCT04353830 |
|  | COM902 | Advanced malignancies | NA | I | 2020  2022 | Recruiting | NCT04354246 |
| CD94/NKG2A | Monalizumab | Hematologic malignancies | NA | I | 2016  2020 | Recruiting | NCT02921685 |
|  |  | Advanced solid tumors | Durvalumab | I/II | 2016  2022 | Active, not recruiting | NCT02671435 |
|  |  | Head and neck neoplasms | Cetuximab  Anti-PD(L)1 | I/II | 2015  2021 | Recruiting | NCT02643550 |
|  |  | Chronic lymphocytic leukemia | NA | I/II | 2015  2019 | Terminated | NCT02557516 |
|  |  | Non-small cell lung cancer | Durvalumab  Oleclumab  AZD6738  DOCETAXEL | II | 2019  2024 | Recruiting | NCT03833440 |

1. Brignone C, Gutierrez M, Mefti F, Brain E, Jarcau R, Cvitkovic F, Bousetta N, Medioni J, Gligorov J, Grygar C, Marcu M, Triebel F. First-line chemoimmunotherapy in metastatic breast carcinoma: combination of paclitaxel and IMP321 (LAG-3Ig) enhances immune responses and antitumor activity. Journal of translational medicine. 2010 Jul 23;8:71. doi:10.1186/1479-5876-8-71.

2. Gutzmer R, Rivoltini L, Levchenko E, Testori A, Utikal J, Ascierto PA, Demidov L, Grob JJ, Ridolfi R, Schadendorf D, Queirolo P, Santoro A, Loquai C, Dreno B, Hauschild A, Schultz E, Lesimple TP, Vanhoutte N, Salaun B, Gillet M, Jarnjak S, De Sousa Alves PM, Louahed J, Brichard VG, Lehmann FF. Safety and immunogenicity of the PRAME cancer immunotherapeutic in metastatic melanoma: results of a phase I dose escalation study. ESMO open. 2016;1(4):e000068. doi:10.1136/esmoopen-2016-000068.

3. Romano E, Michielin O, Voelter V, Laurent J, Bichat H, Stravodimou A, Romero P, Speiser DE, Triebel F, Leyvraz S, Harari A. MART-1 peptide vaccination plus IMP321 (LAG-3Ig fusion protein) in patients receiving autologous PBMCs after lymphodepletion: results of a Phase I trial. Journal of translational medicine. 2014 Apr 12;12:97. doi:10.1186/1479-5876-12-97.
