# Supplementary material for: Anti-Cancer Nanomedicines: A Revolution of Tumor Immunotherapy
Source: Front Immunol. 2020 Dec 21;11:601497. doi: 10.3389/fimmu.2020.601497 (PMC7779686; doi:10.3389/fimmu.2020.601497)
Supplement: Supplementary file 1 [file DataSheet_1.zip › Supplementary Table 2.DOCX]

Supplementary Table S2 Specific indicators of clinical immunotherapy strategies

| Drugs | Targets | Indications | Approval |
| --- | --- | --- | --- |
| BCG Live (TheraCys®) | Vaccine | Carcinoma in situ of the urinary bladder  Primary or recurrent stage Ta and/or T1 papillary tumors | 1990 FDA |
| Sipuleucel-T (Provenge®) | Vaccine | Asymptomatic or minimally symptomatic metastatic castrate-resistant prostate cancer | 2010 FDA |
| Talimogene laherparepvec (T-VEC/Imlygic®) | Vaccine | Local treatment of unresectable cutaneous, subcutaneous, and nodal lesions in patients with melanoma recurrent after initial surgery. | 2015 FDA |
| Aldesleukin | IL-2 | Metastatic renal cell carcinoma,  Metastatic melanoma | 1992 FDA |
| Roferon A | IFN α2a | Hairy cell leukaemia  Chronic myelogenous leukaemia,  AIDS- related Kaposi sarcoma | 1986 FDA |
| Intron A | IFN α2b | Hairy cell leukaemia  Melanoma, Follicular lymphoma  AIDS- related Kaposi sarcoma | 1986 FDA |
| Imiquimod | TNF IL-12  IFN γ | Basal cell carcinoma | 2004 FDA |
| Ipilimumab | CTLA-4 | Melanoma | 2011 FDA |
| Tremelimumab | CTLA-4 | Mesothelioma | 2015 FDA |
| Nivolumab | PD-1 | Melanoma  Non-small cell lung cancer  Renal cell carcinoma  Classical Hodgkin Lymphoma  Head and neck squamous cell cancer  Urothelial carcinoma  Hepatocellular carcinoma  Microsatellite instability-high/mismatch repair deficient metastatic colorectal cancer; | 2014 FDA |
| Pembrolizumab | PD-1 | Melanoma  Non-small cell lung cancer  Head and neck squamous cell cancer,  Classical Hodgkin Lymphoma  Urothelial carcinoma  Microsatellite instability-high cancer  Colorectal cancer  Gastric Cancer | 2014 FDA |
| Cemiplimab | PD-1 | Cutaneous squamous cell carcinoma | 2018 FDA |
| Atezolizumab | PD-L1 | Urothelial carcinoma  Non-small cell lung cancer | 2016 FDA |
| Avelumab | PD-L1 | Merkel cell carcinoma  Urothelial carcinoma | 2017 FDA |
| Durvalumab | PD-L1 | Urothelial carcinoma  Non-small cell lung cancer | 2018 FDA |
| Toripalimab | PD-1 | Melanoma | 2018 NMPA |
| Sintilimab | PD-1 | Classical Hodgkin Lymphoma | 2018 NMPA |
| Elotuzumab | SLAMF7 | Multiple myeloma received one to three prior therapies | 2015 FDA |
| Blinatumomab | CD19/CD3 | Philadelphia chromosome-negative relapsed /refractory B cell precursor acute lymphoblastic leukemia | 2014 FDA |
| brentuximab vedotin | CD30 | Anaplastic large cell lymphoma,  Primary cutaneous anaplastic large cell lymphoma,  Hodgkin lymphoma, CD30+ Mycosis fungoides,  CD30+ Peripheral T-cell lymphoma | 2011 FDA |
| Daratumumab | CD38 | Multiple myeloma | 2015 FDA |
| Obinutuzumab | CD20 | Previously untreated chronic lymphocytic leukemia, Relapsed/refractory follicular lymphoma (FL) | 2016 FDA |

NMPA: National Medical Products Administration; FDA: Food and Drug Administration
