# Supplementary material for: Anti-Cancer Nanomedicines: A Revolution of Tumor Immunotherapy
Source: Front Immunol. 2020 Dec 21;11:601497. doi: 10.3389/fimmu.2020.601497 (PMC7779686; doi:10.3389/fimmu.2020.601497)
Supplement: Supplementary file 1 [file DataSheet_1.zip › Supplementary Table 3.DOCX]

Supplementary Table S3 Nanomedicine for tumor immunotherapy in clinical trials

| Drug | Carrier | Combination | Disease | Status | Phase | Identifier | Reference |
| --- | --- | --- | --- | --- | --- | --- | --- |
| CRLX101 | Cyclodextrin | Camptothecin | Advanced renal cell carcinoma | Completed | I | NCT01625936 | - |
|  |  |  | Rectal cancer | Active, not recruiting | I/II | NCT02010567 | (1) |
|  |  |  | Metastatic castration resistant prostate cancer  Prostate neoplasms | Recruiting | II | NCT03531827 | - |
|  |  |  | Ovarian cancer/Fallopian tube cancer /Primary peritoneal cancer | Completed | II | NCT01652079 | - |
|  |  |  | Non small cell lung cancer  Urothelial cancer | Completed  Recruiting | II  I/II | NCT01380769 NCT02769962 | -  - |
|  |  |  | Advanced gastroesophageal cancer or esophageal cancer | Completed | II | NCT01612546 | - |
| Tecemotide  (L-BLP25) | Liposome | Radiation therapy  Goserelin  Cyclophosphamide | Prostate cancer | Completed | II | NCT01496131 | - |
|  |  | Cyclophosphamide  Chemoradiotherapy | Rectal cancer | Completed | II | NCT01507103 | - |
|  |  | Bevacizumab  Carboplatin  Cyclophosphamide  Paclitaxel  Radiotherapy | Lung cancer | Active, not recruiting | II | NCT00828009 | - |
|  |  | Cyclophosphamide  (Single low dose) | Non small cell lung cancer | Completed | I/II | NCT00960115 | (2) |
|  |  | Cyclophosphamide  (Single low dose) | Carcinoma  Non small cell lung cancer  Lung neoplasms | Completed | II | NCT00157196 | (3) |
|  |  | Cyclophosphamide  (Single low dose) | Lung neoplasms  Carcinoma  Non small cell lung cancer | Completed | II | NCT00157209 | (4) |
|  |  | Cyclophosphamide  (Single low dose) | Non small cell lung cancer | Completed | III | NCT00409188 | (5-7) |
|  |  | Cyclophosphamide  (Single low dose)  (Multiple low dose) | Multiple myeloma | Completed | II | NCT01094548 | - |
|  |  | NA | Colon carcinoma  Rectum carcinoma | Completed | II | NCT01462513 | - |
| Lipo-MERIT | Liposome | RNA antigens induce antigen-specific CD8+ and CD4+ T cell responses | Melanoma | Recruiting | I | NCT02410733 | (8) |
| DPX-0907 | Liposome | NA | Ovarian neoplasms  Breast neoplasms  Prostatic neoplasms | Completed | I | NCT01095848 | (9) |
| DPX- Survivac | Liposome | Cyclophosphamide  (low dose oral) | Ovarian cancer  Fallopian tube cancer  Peritoneal cancer | Completed | I | NCT01416038 | (10) |
|  |  | Cyclophosphamide  Epacadostat | Recurrent epithelial ovarian cancer  Recurrent fallopian tube cancer  Recurrent Peritoneal cancer | Active, not recruiting | I/II | NCT02785250 | - |
|  |  | Pembrolizumab  Cyclophosphamide | Advanced cancer  Ovarian cancer  Primary peritoneal carcinoma  Fallopian tube cancer | Recruiting | II | NCT03029403 | - |
|  |  | Cyclophosphamide | Epithelial ovarian cancer  Fallopian tube cancer  Peritoneal cancer | Active, not recruiting | I | NCT03332576 | - |
|  |  | Cyclophosphamide  Pembrolizumab | Ovarian cancer  Hepatocellular carcinoma  Non small cell lung cancer Bladder cancer  Microsatellite Instability-High | Recruiting | II | NCT03836352 | - |
|  |  | Pembrolizumab  Cyclophosphamide (50mg) | Recurrent adult diffuse large cell lymphoma  Adult refractory diffuse large B-cell lymphoma | Recruiting | II | NCT03349450 | - |
|  |  | Cyclophosphamide | Diffuse large B-cell lymphoma | Active, not recruiting | II | NCT02323230 | - |
| CHP-NY-ESO-1 | Cholesteryl  pullulan | CHP-HER2  CHP-NY-ESO-1 | Esophageal cancer  Lung cancer  Stomach cancer  Breast cancer  Ovarian cancer | Completed | I | NCT00291473 | (11) |
|  |  | NA | Neoplasms | Completed | I | NCT00106158 | (12) |
| IMF-001 | Cholesteryl  pullulan | CHP-NY-ESO-1 | Esophageal cancer | Completed | I | NCT01003808 | (13) |
|  |  |  | Solid tumor | Completed | I | NCT01234012 | - |
| CYT-6091 | Colloidal gold | recombinant human tumor necrosis factor alpha (rhTNF) | Unspecified adult solid tumor  protocol specific | Completed | I | NCT00356980 | (14) |
|  |  | Surgery | Adrenocortical carcinoma  Breast cancer  Colorectal cancer  Gastrointestinal cancer  Kidney cancer  Liver cancer  Melanoma (skin)  Ovarian cancer  Pancreatic cancer  Sarcoma | Completed | I | NCT00436410 | - |
| Oncoquest-L | Proteoliposome | NA | Follicular lymphoma | Not yet recruiting | II | NCT02194751 | - |
| AS15 | Liposome | dHER2  Lapatinib | Metastatic breast cancer | Completed | I/II | NCT00952692 | (15) |
|  |  | MAG-TN3 | Breast neoplasms | Active, not recruiting | I | NCT02364492 | - |
|  |  | MAGE-A3 ASCI | Bladder cancer | Completed | I | NCT01498172 | - |
|  |  | recMAGE-A3 | Urinary bladder neoplasms | Terminated | II | NCT01435356 | (16) |
|  |  | P501 | Neoplasms prostate | Completed | I | NCT00148928 | - |
|  |  | SB719125 (Primary) | Neoplasms breast | Completed | I | NCT00058526 | (17) |
|  |  | Recombinant PRAME protein combined | Non small cell lung cancer | Completed | II | NCT01853878 | - |
|  |  | ecMAGE-A3 Protein | Multiple myeloma | Completed | I | NCT01380145 | (18) |
|  |  | GSK2302025A | Melanoma | Completed | I | NCT01149343 | (19) |
|  |  | recMAGE-A3 | Melanoma | Completed | I | NCT01425749 | (20) |
|  |  | HDIL-2  recMAGE-A3 | Melanoma | Completed | II | NCT01266603 | (21) |
| Lipovaxin MM | Liposome | NA | Melanoma | Completed | I | NCT01052142 | (22) |
| ISCOMATRIX | Liposome | Octavalent HPV / AAHS | Cervical cancer  Vulvar cancer  Vaginal cancer | Completed | I | NCT00851643 | - |
|  |  | NY-ESO-1 | Melanoma | Completed | II | NCT00199901 | - |
|  |  | NY-ESO-1  Cyclophosphamide | Melanoma | Completed | II | NCT00518206 | (23, 24) |
|  |  | Celebrex  Tumor cell vaccine | Mesolthelioma  Esophageal cancer  Lung cancer  Thoracic sarcomas  Thymoma | Terminated | I | NCT01258868 | - |
|  |  | Epigenetically Modified Autologous Tumor  Cyclophosphamide  Celecoxib | Sarcoma  Melanoma  Epithelial malignancies  Pleural malignancy | Terminated | I | NCT01341496 | - |
| JVRS-100 | Liposome | NA | Leukemia | Completed | I | NCT00860522 | (25, 26) |
| Melan-A VLPs | VLPs | CYT004-MelQbG10 | Malignant melanoma | Completed | I/II | NCT00306566 | - |
|  |  | CYT004-MelQbG10 | Melanoma | Completed | I/II | NCT00306514 | - |
|  |  | CYT004-MelQbG10 | Malignant melanoma | Completed | II | NCT00306553 | - |
|  |  | CYT004-MelQbG10 + Montanide  CYT004-MelQbG10 + Montanide + Imiquimod  CYT004-MelQbG10 + Imiquimod  CYT004-MelQbG10 intra nodal injection | Malignant melanoma | Completed | II | NCT00651703 | (27) |
|  |  | Melan-A VLP vaccine, IMP321 adjuvant  Adoptive immunotherapy  Therapeutic autologous lymphocytes  Cyclophosphamide  Fludarabine phosphate | Melanoma (skin) | Completed | I | NCT00324623 | (28) |
| ONT-10 | PET Lipid A | Glycolipopeptide antigen | Solid tumors | Completed | I | NCT01556789 | - |
|  |  |  | Advanced breast carcinoma  Advanced ovarian carcinoma | Completed | I | NCT02270372 | - |
| ZYC300 | PLG microparticles | plasmid DNA of CYP1B1 | Breast cancer  Ovarian cancer  Prostate cancer  Colon cancer  Renal cancer | Completed | I | NCT00381173 | (29) |
| Dex2 | dendritic cell-derived exosomes (Dex) | tumor antigen | Non small cell lung cancer | Completed | II | NCT01159288 | (30) |
| DRibbles | Autophagosome | Dribble vaccine | Carcinoma  Non small cell lung cancer | Not yet recruiting | I | NCT03057340 | (31,  32-34) |
|  | Autophagosome | DRibble Vaccine  HPV Vaccinations  Cyclophosphamide | Adenocarcinoma of the prostate | Completed | I | NCT02234921 | - |
|  | Autophagosome | DRibble vaccine  GM-CSF | Non small cell lung cancer | Completed | I | NCT00850785 | - |
|  | Autophagosome | Cyclophosphamide  DRibble vaccine  Imiquimod  GM-CSF  HPV vaccine | Carcinoma  Non small cell lung cancer | Completed | II | NCT01909752 | - |
| WDVAX | Tumor lysate | GM-CSF  CpG. | Melanoma | Active, not recruiting | I | NCT01753089 | - |

REFERENCES

1. Sanoff HK, Moon DH, Moore DT, Boles J, Bui C, Blackstock W, O'Neil BH, Subramaniam S, McRee AJ, Carlson C, Lee MS, Tepper JE, Wang AZ. Phase I/II trial of nano-camptothecin CRLX101 with capecitabine and radiotherapy as neoadjuvant treatment for locally advanced rectal cancer. Nanomedicine-Uk. 2019 Jun;18:189-195. doi:10.1016/j.nano.2019.02.021.

2. Katakami N, Hida T, Nokihara H, Imamura F, Sakai H, Atagi S, Nishio M, Kashii T, Satouchi M, Helwig C, Watanabe M, Tamura T. Phase I/II study of tecemotide as immunotherapy in Japanese patients with unresectable stage III non-small cell lung cancer. Lung cancer. 2017 Mar;105:23-30. doi:10.1016/j.lungcan.2017.01.007.

3. Butts C, Murray RN, Smith CJ, Ellis PM, Jasas K, Maksymiuk A, Goss G, Ely G, Beier F, Soulieres D. A multicenter open-label study to assess the safety of a new formulation of BLP25 liposome vaccine in patients with unresectable stage III non-small-cell lung cancer. Clin Lung Cancer. 2010 Nov 1;11(6):391-5. doi:10.3816/CLC.2010.n.101.

4. Butts C, Maksymiuk A, Goss G, Soulieres D, Marshall E, Cormier Y, Ellis PM, Price A, Sawhney R, Beier F, Falk M, Murray N. Updated survival analysis in patients with stage IIIB or IV non-small-cell lung cancer receiving BLP25 liposome vaccine (L-BLP25): phase IIB randomized, multicenter, open-label trial. Journal of cancer research and clinical oncology. 2011 Sep;137(9):1337-42. doi:10.1007/s00432-011-1003-3.

5. Butts C, Socinski MA, Mitchell PL, Thatcher N, Havel L, Krzakowski M, Nawrocki S, Ciuleanu TE, Bosquee L, Trigo JM, Spira A, Tremblay L, Nyman J, Ramlau R, Wickart-Johansson G, Ellis P, Gladkov O, Pereira JR, Eberhardt WE, Helwig C, Schroder A, Shepherd FA, team St. Tecemotide (L-BLP25) versus placebo after chemoradiotherapy for stage III non-small-cell lung cancer (START): a randomised, double-blind, phase 3 trial. The Lancet Oncology. 2014 Jan;15(1):59-68. doi:10.1016/S1470-2045(13)70510-2.

6. Mitchell P, Thatcher N, Socinski MA, Wasilewska-Tesluk E, Horwood K, Szczesna A, Martin C, Ragulin Y, Zukin M, Helwig C, Falk M, Butts C, Shepherd FA. Tecemotide in unresectable stage III non-small-cell lung cancer in the phase III START study: updated overall survival and biomarker analyses. Ann Oncol. 2015 Jun;26(6):1134-42. doi:10.1093/annonc/mdv104.

7. Rossmann E, Osterborg A, Lofvenberg E, Choudhury A, Forssmann U, von Heydebreck A, Schroder A, Mellstedt H. Mucin 1-specific active cancer immunotherapy with tecemotide (L-BLP25) in patients with multiple myeloma: an exploratory study. Human vaccines & immunotherapeutics. 2014;10(11):3394-408. doi:10.4161/hv.29918.

8. Jabulowsky RA, Loquai C, Derhovanessian E, Mitzel-Rink H, Utikal J, Hassel J, Kaufmann R, Pinter A, Diken M, Gold M, Heesen L, Schreeb KH, Schwarck-Kokarakis D, Kreiter S, Gaiser MR, Jager D, Grabbe S, Tureci O, Sahin U. A first-in-human phase I/II clinical trial assessing novel mRNA-lipoplex nanoparticles encoding shared tumor antigens for immunotherapy of malignant melanoma. Ann Oncol. 2018 Oct;29:439-439. English.

9. Berinstein NL, Karkada M, Morse MA, Nemunaitis JJ, Chatta G, Kaufman H, Odunsi K, Nigam R, Sammatur L, MacDonald LD, Weir GM, Stanford MM, Mansour M. First-in-man application of a novel therapeutic cancer vaccine formulation with the capacity to induce multi-functional T cell responses in ovarian, breast and prostate cancer patients. Journal of translational medicine. 2012 Aug 3;10. English. doi:Artn 15610.1186/1479-5876-10-156.

10. Berinstein NL, Karkada M, Oza AM, Odunsi K, Villella JA, Nemunaitis JJ, Morse MA, Pejovic T, Bentley J, Buyse M, Nigam R, Weir GM, MacDonald LD, Quinton T, Rajagopalan R, Sharp K, Penwell A, Sammatur L, Burzykowski T, Stanford MM, Mansour M. Survivin-targeted immunotherapy drives robust polyfunctional T cell generation and differentiation in advanced ovarian cancer patients. Oncoimmunology. 2015;4(8). English. doi:ARTN e1026529 10.1080/2162402X.2015.1026529.

11. Aoki M, Ueda S, Nishikawa H, Kitano S, Hirayama M, Ikeda H, Toyoda H, Tanaka K, Kanai M, Takabayashi A, Imai H, Shiraishi T, Sato E, Wada H, Nakayama E, Takei Y, Katayama N, Shiku H, Kageyama S. Antibody responses against NY-ESO-1 and HER2 antigens in patients vaccinated with combinations of cholesteryl pullulan (CHP)-NY-ESO-1 and CHP-HER2 with OK-432. Vaccine. 2009 Nov 16;27(49):6854-6861. English. doi:10.1016/j.vaccine.2009.09.018.

12. Wada H, Sato E, Uenaka A, Isobe M, Kawabata R, Nakamura Y, Iwae S, Yonezawa K, Yamasaki M, Miyata H, Doki Y, Shiku H, Jungbluth AA, Ritter G, Murphy R, Hofftnan EW, Old LJ, Monden M, Nakayama E. Analysis of peripheral and local anti-tumor immune response in esophageal cancer patients after NY-ESO-1 protein vaccination. International Journal of Cancer. 2008 Nov 15;123(10):2362-2369. English. doi:10.1002/ijc.23810.

13. Kageyama S, Wada H, Muro K, Niwa Y, Ueda S, Miyata H, Takiguchi S, Sugino SH, Miyahara Y, Ikeda H, Imai N, Sato E, Yamada T, Osako M, Ohnishi M, Harada N, Hishida T, Doki Y, Shiku H. Dose-dependent effects of NY-ESO-1 protein vaccine complexed with cholesteryl pullulan (CHP-NY-ESO-1) on immune responses and survival benefits of esophageal cancer patients. Journal of translational medicine. 2013 Oct 5;11. English. doi:Artn 246 10.1186/1479-5876-11-246.

14. Libutti SK, Paciotti GF, Byrnes AA, Alexander HR, Gannon WE, Walker M, Seidel GD, Yuldasheva N, Tamarkin L. Phase I and Pharmacokinetic Studies of CYT-6091, a Novel PEGylated Colloidal Gold-rhTNF Nanomedicine. Clinical Cancer Research. 2010 Dec 15;16(24):6139-6149. English. doi:10.1158/1078-0432.CCR-10-0978.

15. Hamilton E, Blackwell K, Hobeika AC, Clay TM, Broadwater G, Ren XR, Chen W, Castro H, Lehmann F, Spector N, Wei J, Osada T, Lyerly HK, Morse MA. Phase 1 clinical trial of HER2-specific immunotherapy with concomitant HER2 kinase inhibition [corrected]. Journal of translational medicine. 2012 Feb 10;10:28. doi:10.1186/1479-5876-10-28.

16. Colombel M, Heidenreich A, Martinez-Pineiro L, Babjuk M, Korneyev I, Surcel C, Yakovlev P, Colombo R, Radziszewski P, Witjes F, Schipper R, Mulders P, Witjes WP. Perioperative chemotherapy in muscle-invasive bladder cancer: overview and the unmet clinical need for alternative adjuvant therapy as studied in the MAGNOLIA trial. European urology. 2014 Mar;65(3):509-11. doi:10.1016/j.eururo.2013.10.056.

17. Limentani SA, Campone M, Dorval T, Curigliano G, de Boer R, Vogel C, White S, Bachelot T, Canon JL, Disis M, Awada A, Berliere M, Amant F, Levine E, Burny W, Callegaro A, de Sousa Alves PM, Louahed J, Brichard V, Lehmann FF. A non-randomized dose-escalation Phase I trial of a protein-based immunotherapeutic for the treatment of breast cancer patients with HER2-overexpressing tumors. Breast cancer research and treatment. 2016 Apr;156(2):319-30. doi:10.1007/s10549-016-3751-x.

18. Cohen AD, Lendvai N, Nataraj S, Imai N, Jungbluth AA, Tsakos I, Rahman A, Mei AH, Singh H, Zarychta K, Kim-Schulze S, Park A, Venhaus R, Alpaugh K, Gnjatic S, Cho HJ. Autologous Lymphocyte Infusion Supports Tumor Antigen Vaccine-Induced Immunity in Autologous Stem Cell Transplant for Multiple Myeloma. Cancer Immunol Res. 2019 Apr;7(4):658-669. doi:10.1158/2326-6066.CIR-18-0198.

19. Gutzmer R, Rivoltini L, Levchenko E, Testori A, Utikal J, Ascierto PA, Demidov L, Grob JJ, Ridolfi R, Schadendorf D, Queirolo P, Santoro A, Loquai C, Dreno B, Hauschild A, Schultz E, Lesimple TP, Vanhoutte N, Salaun B, Gillet M, Jarnjak S, De Sousa Alves PM, Louahed J, Brichard VG, Lehmann FF. Safety and immunogenicity of the PRAME cancer immunotherapeutic in metastatic melanoma: results of a phase I dose escalation study. ESMO open. 2016;1(4):e000068. doi:10.1136/esmoopen-2016-000068.

20. Slingluff CL, Jr., Petroni GR, Olson WC, Smolkin ME, Chianese-Bullock KA, Mauldin IS, Smith KT, Deacon DH, Varhegyi NE, Donnelly SB, Reed CM, Scott K, Galeassi NV, Grosh WW. A randomized pilot trial testing the safety and immunologic effects of a MAGE-A3 protein plus AS15 immunostimulant administered into muscle or into dermal/subcutaneous sites. Cancer immunology, immunotherapy : CII. 2016 Jan;65(1):25-36. doi:10.1007/s00262-015-1770-9.

21. McQuade JL, Homsi J, Torres-Cabala CA, Bassett R, Popuri RM, James ML, Vence LM, Hwu WJ. A phase II trial of recombinant MAGE-A3 protein with immunostimulant AS15 in combination with high-dose Interleukin-2 (HDIL2) induction therapy in metastatic melanoma. Bmc Cancer. 2018 Dec 19;18(1):1274. doi:10.1186/s12885-018-5193-9.

22. Gargett T, Abbas MN, Rolan P, Price JD, Gosling KM, Ferrante A, Ruszkiewicz A, Atmosukarto IIC, Altin J, Parish CR, Brown MP. Phase I trial of Lipovaxin-MM, a novel dendritic cell-targeted liposomal vaccine for malignant melanoma. Cancer immunology, immunotherapy : CII. 2018 Sep;67(9):1461-1472. doi:10.1007/s00262-018-2207-z.

23. Nicholaou T, Ebert LM, Davis ID, McArthur GA, Jackson H, Dimopoulos N, Tan B, Maraskovsky E, Miloradovic L, Hopkins W, Pan L, Venhaus R, Hoffman EW, Chen W, Cebon J. Regulatory T-cell-mediated attenuation of T-cell responses to the NY-ESO-1 ISCOMATRIX vaccine in patients with advanced malignant melanoma. Clinical cancer research : an official journal of the American Association for Cancer Research. 2009 Mar 15;15(6):2166-73. doi:10.1158/1078-0432.CCR-08-2484.

24. Klein O, Davis ID, McArthur GA, Chen L, Haydon A, Parente P, Dimopoulos N, Jackson H, Xiao K, Maraskovsky E, Hopkins W, Stan R, Chen W, Cebon J. Low-dose cyclophosphamide enhances antigen-specific CD4(+) T cell responses to NY-ESO-1/ISCOMATRIX vaccine in patients with advanced melanoma. Cancer immunology, immunotherapy : CII. 2015 Apr;64(4):507-18. doi:10.1007/s00262-015-1656-x.

25. Kulkarni JA, Cullis PR, van der Meel R. Lipid Nanoparticles Enabling Gene Therapies: From Concepts to Clinical Utility. Nucleic acid therapeutics. 2018 Jun;28(3):146-157. doi:10.1089/nat.2018.0721.

26. Tyagi P, Santos JL. Macromolecule nanotherapeutics: approaches and challenges. Drug discovery today. 2018 May;23(5):1053-1061. doi:10.1016/j.drudis.2018.01.017.

27. Goldinger SM, Dummer R, Baumgaertner P, Mihic-Probst D, Schwarz K, Hammann-Haenni A, Willers J, Geldhof C, Prior JO, Kundig TM, Michielin O, Bachmann MF, Speiser DE. Nano-particle vaccination combined with TLR-7 and -9 ligands triggers memory and effector CD8(+) T-cell responses in melanoma patients. European journal of immunology. 2012 Nov;42(11):3049-61. doi:10.1002/eji.201142361.

28. Romano E, Michielin O, Voelter V, Laurent J, Bichat H, Stravodimou A, Romero P, Speiser DE, Triebel F, Leyvraz S, Harari A. MART-1 peptide vaccination plus IMP321 (LAG-3Ig fusion protein) in patients receiving autologous PBMCs after lymphodepletion: results of a Phase I trial. Journal of translational medicine. 2014 Apr 12;12:97. doi:10.1186/1479-5876-12-97.

29. Gribben JG, Ryan DP, Boyajian R, Urban RG, Hedley ML, Beach K, Nealon P, Matulonis U, Campos S, Gilligan TD, Richardson PG, Marshall B, Neuberg D, Nadler LM. Unexpected association between induction of immunity to the universal tumor antigen CYP1B1 and response to next therapy. Clinical cancer research : an official journal of the American Association for Cancer Research. 2005 Jun 15;11(12):4430-6. doi:10.1158/1078-0432.CCR-04-2111.

30. Pitt JM, Andre F, Amigorena S, Soria JC, Eggermont A, Kroemer G, Zitvogel L. Dendritic cell-derived exosomes for cancer therapy. The Journal of clinical investigation. 2016 Apr 1;126(4):1224-32. doi:10.1172/JCI81137.

31. Ye W, Xing Y, Paustian C, van de Ven R, Moudgil T, Hilton TL, Fox BA, Urba WJ, Zhao W, Hu HM. Cross-presentation of viral antigens in dribbles leads to efficient activation of virus-specific human memory T cells. Journal of translational medicine. 2014 Apr 16;12:100. doi:10.1186/1479-5876-12-100.

32. Xue M, Fan F, Ding L, Liu J, Su S, Yin P, Cao M, Zhao W, Hu HM, Wang L. An autophagosome-based therapeutic vaccine for HBV infection: a preclinical evaluation. Journal of translational medicine. 2014 Dec 20;12:361. doi:10.1186/s12967-014-0361-4.

33. Zhou M, Li W, Wen Z, Sheng Y, Ren H, Dong H, Cao M, Hu HM, Wang LX. Macrophages enhance tumor-derived autophagosomes (DRibbles)-induced B cells activation by TLR4/MyD88 and CD40/CD40L. Exp Cell Res. 2015 Feb 15;331(2):320-30. doi:10.1016/j.yexcr.2014.10.015.

34. Ren H, Zhao S, Li W, Dong H, Zhou M, Cao M, Hu HM, Wang LX. Therapeutic antitumor efficacy of B cells loaded with tumor-derived autophagasomes vaccine (DRibbles). J Immunother. 2014 Oct;37(8):383-93. doi:10.1097/CJI.0000000000000051.
