# Supplementary material for: Anti-Cancer Nanomedicines: A Revolution of Tumor Immunotherapy
Source: Front Immunol. 2020 Dec 21;11:601497. doi: 10.3389/fimmu.2020.601497 (PMC7779686; doi:10.3389/fimmu.2020.601497)
Supplement: Supplementary file 1 [file DataSheet_1.zip › Supplementary Table 4.DOCX]

Supplementary Table S4 Clinical applied nanomedicine for tumor therapy

| Drug | Carrier | Anti-tumor agents | Indications |
| --- | --- | --- | --- |
| Nab-paclitaxel  (Abraxane ®) | Albumin | Paclitaxel | Metastatic breast cancer (with anthracycline)  NSCLC (with carboplatin)  Metastatic adenocarcinoma of pancreas (with gemcitabine) |
| Doxorubicin hydrochloride liposome (DOXIL ®) | Pegylated liposomal | Doxorubicin | Ovarian cancer  AIDS-Related Kaposi's Sarcoma  Multiple myeloma |
| Doxorubicin hydrochloride liposome (Caelyx®) | Pegylated liposomal | Doxorubicin | AIDS-Related Kaposi's Sarcoma with low CD4 counts  Metastatic breast cancer  Advanced ovarian cancer  Multiple myeloma |
| Pegylated liposomal doxorubicin (Lipo-Dox®) | Pegylated liposomal | Doxorubicin | AIDS-Related Kaposi's Sarcoma with low CD4 counts  Metastatic breast cancer  Advanced ovarian cancer  Multiple myeloma |
| Non-pegylated liposomal doxorubicin (Myocet®) | Non-pegylated liposomal | Doxorubicin | Breast cancer |
| Vincristine sulfate liposome  (Marqibo ®) | Liposome | Vincristine | Adult patients with Philadelphia chromosome–negative (Ph-) acute lymphoblastic leukemia |
| Irinotecan liposome  (Onivyde®) | Liposome | Irinotecan | Metastatic pancreatic cancer |
| DaunoXome® | Liposome | Daunorubicin | HIV-associated Kaposi's sarcoma |
| DepoCyt® | Liposome | Cytarabine | Lymphomatous meningitis |
| Genexol-PM® | PEG-b-PLA micelle | Paclitaxel | Breast cancer  Lung cancer,  Ovarian cancer |
| NanoTherm® | Iron oxide nanoparticle |  | Thermal ablation |
| Mepact® | Liposomal muramyl tripeptide | Mifamurtide | Osteosarcoma |
| Pegaspargase  Oncaspar® | PEG | Aspargase | Acute lymphoblastic leukemia (ALL) |
| Denileukin diftitox  Ontak® | Fragments of diphtheria toxin linked to human IL-2 | IL-2 receptor  Diphtheria toxin | CD25^+^ T-cell lymphoma |
| SMANCS® |  | Zinostatin stimalamer | Liver cancer  Renal cancer |
